# UAV-Based Secure Data Communication: Multilevel Authentication Perspective

**DOI:** 10.3390/s24030996

**Published:** 2024-02-03

**Authors:** Abdullah Aljumah

**Affiliations:** College of Computer Engineering and Sciences, Prince Sattam Bin Abdulaziz University, Al-Kharj 11942, Saudi Arabia; aljumah@psau.edu.sa

**Keywords:** UAV, security, authentication

## Abstract

An Internet of Things (IoT) system for managing and coordinating unmanned aerial vehicles (UAVs) has revolutionized the industrial sector. The largest issue with the design of the Internet of UAVs (IoUAV) is security. Conspicuously, the novel contribution of the proposed work is to develop a layered authentication approach to facilitate safe IoUAV communication. Specifically, four modules, including the pre-deployment module, user registration module, login module, and authentication module, form the basis of security analysis. In the proposed technique, UAVs are added to the IoUAV registry. The next step is the user registration module, where people are registered with the UAV so they may access the information in real time. In the login module, the user connects with the server for data transmission. Finally, in the authentication module, all entities, including users, servers, and UAVs, are authenticated to ensure secure data communication. The proposed method achieves peak performance as compared to the state-of-the-art techniques in terms of statistical parameters of latency (3.255s), throughput (90.15%), and packet loss (8.854%).

## 1. Introduction

As the Internet of Things (IoT) continues to develop, communication and data sharing among various devices become possible. Unmanned aerial vehicles (UAVs), also known as drones, are utilized in IoT smart gadgets to gather data from multiple sources in sectors such as military, agriculture, parking, and more [1,2]. UAVs can be categorized into multirotor systems and fixed-wing systems, with the latter being more commonly used [3,4]. Fixed-wing aircraft generate lift through static wings and forward velocity, while rotorcraft, including multirotor systems, generate lift using rotating wings. Hybrid systems combine features from both designs [5]. UAV technology has a wide range of applications, including film-making, animal monitoring, infrastructure inspection, agriculture, airspace control, exploration, and conservation. UAVs are also being utilized in global logistics for on-demand delivery and long-distance transportation [6]. The Internet of UAVs (IoUAV) is a network that connects unmanned aerial vehicles used for complex missions. It provides control, coordination, routing services, and various other functionalities for UAVs [7]. The IoUAV enables UAVs to detect physical phenomena through built-in sensors and capture images and videos through onboard cameras. The collected data are then transmitted to the UAV’s control box using wireless technologies like WiFi [8,9]. However, it is crucial to address security concerns when it comes to UAV technology. UAVs are vulnerable to attacks such as manipulation and interception, which can compromise their integrity and authenticity [10,11,12]. Ensuring security in communication between ground sensors and UAVs is essential, including aspects like non-repudiation, integrity, secrecy, and authentication [13,14]. While UAVs equipped with sensor data offer valuable solutions for the IoUAV network, it is important to manage the associated security risks. Some of the major risks considered in the current context include the following:

1. Considering that the erasing cipher is not readily cracked, the reliance on a protective mechanism with high computational security is a major consideration.
2. Enormous datasets can be securely encrypted and decrypted by UAVs with less computational power.
3. The third issue is that the servers store sensitive IoUAV data, making them easier for other hackers to access.

Possible countermeasures to these attacks include algorithms for monitoring UAVs, location detection protocols, and other similar technologies, as well as the jamming of UAVs that are deemed susceptible [15]. Information gathered in real time is the basis of most applications that use IoUAV infrastructure. As a result, it is easy to grasp how a particular zone might provide actual UAV data [16]. This should be doable if users are provided access to real-time data collected by the UAVs. The network becomes more complex when third-party services are taken into account, and the IoUAV organization may face cyber risks. A large number of IoUAV applications keep an eye on cyber vulnerabilities to gather analysis [17]. The investigation began with the IoUAV cybersecurity evaluation. UAV tracking helps to make the airspace around us safer by reducing the likelihood of accidents and improving traffic flow by keeping UAVs out of congested areas. There are several challenges associated with implementing IoUAV [18]. In addition, a major obstacle to safely accessing the UAV’s resources via authentication is security. A major problem with IoUAV is the communication quality. The availability, authenticity, reliability, and secrecy of UAVs are all impacted by security flaws. Conspicuously, easing security problems including illegal affiliation, malicious control, unauthorized access, and other forms of harmful attack is a priority. Typically, message security is provided using cryptographic algorithms [19]. So, to authenticate users with the UAV and obtain access to information about a certain fly zone, the current article suggests a multilevel authentication approach. To provide safe authentication, the suggested technique goes through four modules, including pre-deployment, user registration module, login module, and authentication module. Before each UAV’s IoT deployment, the servers must first register it. The next step is to utilize the user registration phase to get external users registered to view the UAV’s real-time data. The next step in obtaining access to the UAVs is the login process. A user’s login is successful, and the authentication step begins to ensure that the server, UAV, and user can communicate securely.

### Novel Contributions

1. A lightweight user authentication approach, featuring a layered authentication technique, was created as a significant contribution. This method was employed to establish a secure connection between the UAVs. The suggested approach utilizes encryption and bitwise XOR operations to ensure secure communication.
2. There are four modules to evaluate security, including the pre-deployment module, user registration module, login module, and authentication module.
3. Several layers of authentication are used. The first analyzer is for the server to verify the user’s identity before approving any communications. The next layer is to modify the hashing algorithm to make it resistant to different types of attacks. To obtain data from the UAV, the suggested approach made use of several authentication mechanisms, which contribute to secure communication with little latency, high throughput, and low packet loss.
4. A latency of 0.099 s, throughput of 62.855 bps, and packet loss of 2.52012 s were registered as the performance metrics for the suggested approach.

Paper Organization: The rest of the paper is organized in different sections. Section 2 discusses some of the related works in the current domain of study. Section 3 discusses the proposed technique for secure authentication. Experimental validation is performed in Section 4. Finally, Section 5 concludes the paper with future research directions.

## 2. Literature Review

This section discusses different related works in the current study domain. Saini et al. [20] developed cryptography protocols for data protection. In this case, the protocols reduced computation overhead in the object to minimize the time it took to establish the shared key between the object and the UAVs. Several items had privacy-sensitive data communicated, and data were collected from massive UAVs using the developed protocol. However, smart cities that make use of a variety of mobile devices with variable capacity were unable to use this approach. Using the hash function as its foundation, Nguyen et al. [21] developed a swarm flight message authentication technique. To activate the UAVs more quickly and with less drain on the battery, the hash function was used. However, the solution failed to counter the denial-of-service attack, even if it allowed for message authentication and communication across UAV groups. Tsou et al. [22] created a security system to carry out signature revocation during network exploration and search. The technique was used for the implementation of several elliptic curve group signature systems. While the approach performed well in fixing the synchronization problems, it did struggle with weak signal revocation. To achieve safe autonomous flying, Restivo et al. [23] developed a system for controlling the multirotor UAV. This approach allows for real-time operation. The ground controller was built with a certain constant in mind to help with security. This strategy ensured that the necessary precautions would be taken to prevent counterfeit attacks while verifying the authenticity or concealment of the UAV. To provide encryption and handle keys, the authors devised attribute-based encryption. To ensure safety, the strategy relied on UAVs to bolster the sophisticated networks. To strengthen security, the approach was unable to provide unified security access requirements. Because of the insertion of UAVs, the approach also encountered several security challenges and attack vulnerabilities. A formal verification and modeling framework based on UPPAAL was developed by Nasr et al. [24]. The procedure programmed the microcontroller units of autonomous cars and verified that the data received from the sensors and communication modules generated valid packets. With the computational costs and functional aspects in mind, Zhang et al. [25] created an authentication mechanism. The approach provided enhanced security for the IoUAV infrastructure by using sophisticated functionalities. On the other hand, the approach failed to identify suspicious attacks and provide answers to potential obstacles. A user authentication technique for accessing UAV data was developed by Li et al. [26]. To protect against these known threats, the approach made use of the AVISPA tool, which validates Internet security protocols and applications automatically. The model’s viability was impacted by the significant packet loss, yet the approach was effective with various settings and provided improved functionality and safety features. When it comes to safe communication between UAVs, Khoshafa et al. [19] utilized a two-pronged approach. At first, a plethora of safety regulations were put into place to identify malicious actions to find hostile UAVs. After that, to prevent the regular UAV from receiving false data from hostile UAVs, a three-step negotiation was used to identify a mobile agent for each UAV. Secure communications among UAVs are guided by a two-stage process. For secure data communication among users to transfer information between UAVs in a 5G network, Yin et al. [27] created an elliptic curve cryptographic-based source authentication scheme. For secure communication between UAVs, Lan et al. [28] created an access control system based on blockchain. The authentication and access control phases are the two main components of the proposed approach. Using the secret key, the communicating entities exchange information in this technique. There are four stages to the suggested strategy’s implementation of mutual authentication. In addition to processing communication without loss, secure communication also limits unauthorized data access. To protect the detected data from attacks, current timestamps are used. Based on the aforementioned aspects, Table 1 has been formulated to depict the novel aspects of the proposed model.

## 3. Proposed Model

**Figure 1:**
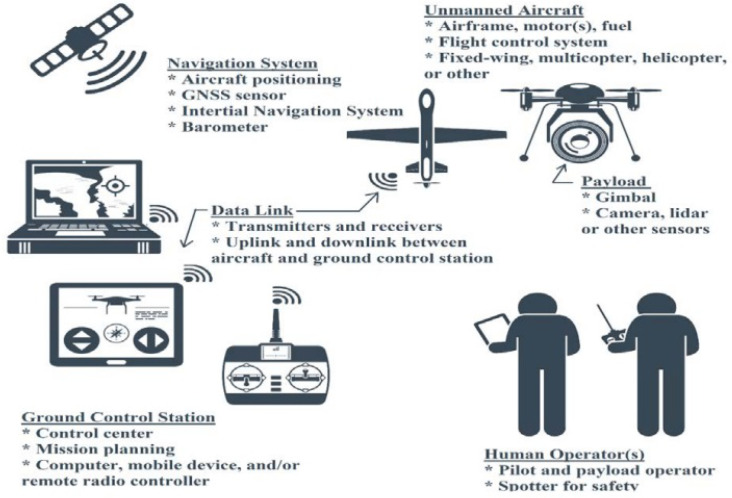
Generalized IoT-UAV model.

The IoUAV system offers various applications for UAVs, including search and rescue, traffic monitoring, and package delivery. Figure 1 illustrates the IoUAV system model. The main objective is to enable UAV flight management through cellular network integration. The model consists of four key modules, namely user module, server module, UAV module, and control room module. In the current scenario, the UAVs can transmit essential data to the control center while operating within a designated fly zone. The UAV’s built-in sensors capture data on physical phenomena such as hazardous gases and temperature, which are then transmitted to the UAV’s control box via WiFi. The UAV establishes communication with a central control center over a network. To ensure secure data access, authentication is employed whenever users request data. Given the potential for various attacks in an IoUAV environment (e.g., brute-force attacks, hacking, or man-in-the-middle attacks), a secure authentication method is crucial for effective communication among users, UAVs, and servers.

### 3.1. Proposed Strategy

The proposed authentication approach utilized in an IoUAV setting is discussed in detail. The method involves positioning various UAVs in different zones of the desired field to send data to the server. An external user, v, can access the system through network access. For real-time data collection, secure authentication among the user, server, and UAVs is crucial. After mutual authentication, the user and UAV can communicate securely, with current timestamps used to protect the data from attacks. The suggested model uses a lightweight approach for validation employing hashing functions, encryption, and bitwise XOR operations. The proposed paradigm undergoes four stages of mutual authentication during login, after authentication, and before deployment. This technology enables remote UAV operation and real-time data acquisition over the Internet. The platform provides users with access to multiple UAVs that can be launched via an Internet connection. IoUAV has broad-ranging applications, including security monitoring, disaster recovery, and more. Several symbolic representation used in the paper is depicted in Table 2.

#### 3.1.1. Verification Process

Users and UAVs are the two main parts of the IoUAV system. The server verifies the users’ identities before allowing the users to communicate with the UAVs.

1. The UAV system includes data about the UAVs that are updated in real time. Through web-related services, client apps can access the UAVs’ resources.
2. Data about the individuals who will be granted access to the UAVs according to their permissions are stored in the user module.
3. One of the most essential building blocks for starting conversations in a network is the communication block.

Providing easy access to the UAVs so that servers can monitor them is a primary goal of the IoUAV system. The procedure that the IoUAV system follows is discussed ahead:

1. The user authenticates the UAV by transmitting their identity, and the server verifies it.
2. The login procedure is activated to reply to the service requests made by the user.
3. To confirm the user, authentication is carried out between the user and the UAV.
4. After user authentication, data about the user’s location are sent to the service engine.
5. The service processor then uses the database to retrieve more information about the service.

An entire procedure is depicted in Algorithm 1. Below, an outline of the four modules that comprise the security analysis is discussed.
**Algorithm 1** Registration and Verification Procedure**Require:** UAV data, Server Data, Server Storage**Ensure:** UAV registration and verification *Begin*: *Pre-deployment Module* **while** New-UAV = Null **do**  Compute Relative Identity Value of New-UAV = ;  Compute the Server Identity value for New-UAV;  Compute Polynomial Identity value of New-UAV;  Store (Relative Identity Value, Server Identity value, Polynomial Identity value) to Server Storage; **end while** *Begin*: *Verification* **if** New-UAV value== Server Data **then**  Generate Fingerprint of New-UAV;  Store Fingerprint in Server Storage; **else**  Generate Alert (“UAV not verified”); **end if**

#### 3.1.2. Initial Deployment Module

**Figure 2:**
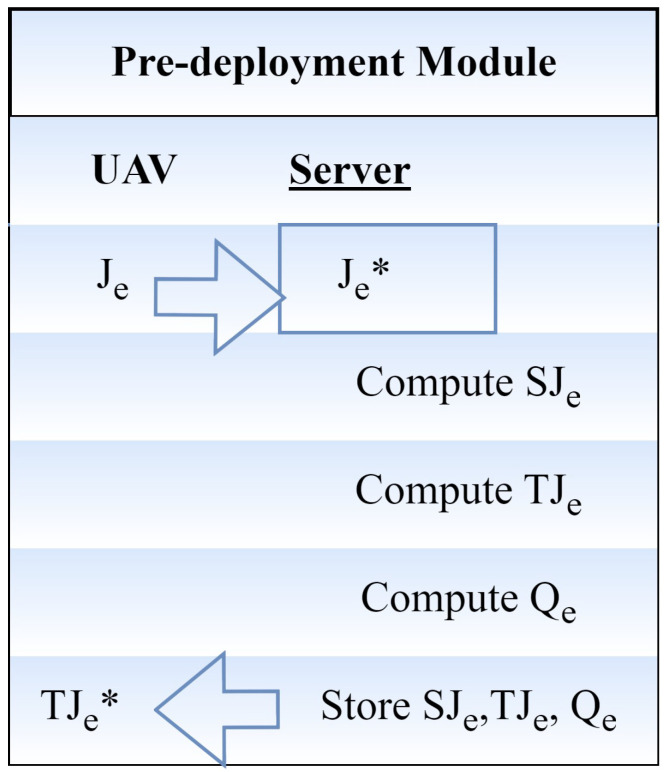
Registration module.

Before any UAV may be used in the IoUAV infrastructure, it must first be registered with the server during the pre-deployment process. The pre-deployment module is shown as mathematical pseudo-code in Figure 2. In this case, the pseudo-identity is computed as follows. For each UAV e_j_, server A selects an identity J_e_ and a message n.

(1) $$SJ_{e}=I\left(J_{e}*\right)Mod\left(n\right)$$

SJ_e_ is the relative UAV identification, I(.) is the hashing function, n is the secret message, and J_e_ is the received UAV identity on the server side. To start the session, server T calculates a session identification, TJ_e_. One way to obtain the session ID is to use

(2) $$TJ_{e}=F\left(J_{e}*\right)I\left[q<>S_{e}\right]$$

where F(.) denotes the encryption and S_e_ denotes the registration timestamp. The Chebyshev polynomial equation is chosen by server A for pair-wise keys with two adjacent UAVs. It is written as

(3) $$Q_{e}=y\left(16y^{4}-20y^{2}+5\right)$$

In this case, Q_e_ is the UAV’s polynomial identity.

(4) $$y=I(TJ_{e}<>J_{a}<>SJ_{e})$$

where the server’s identification is denoted by J_a_. Once the UAV is deployed in the deployment region, the data SJ_e_, TJ_e_, Q_e_ are stored in the UAV’s data storage device by server A.

#### 3.1.3. Registration Module

**Figure 3:**
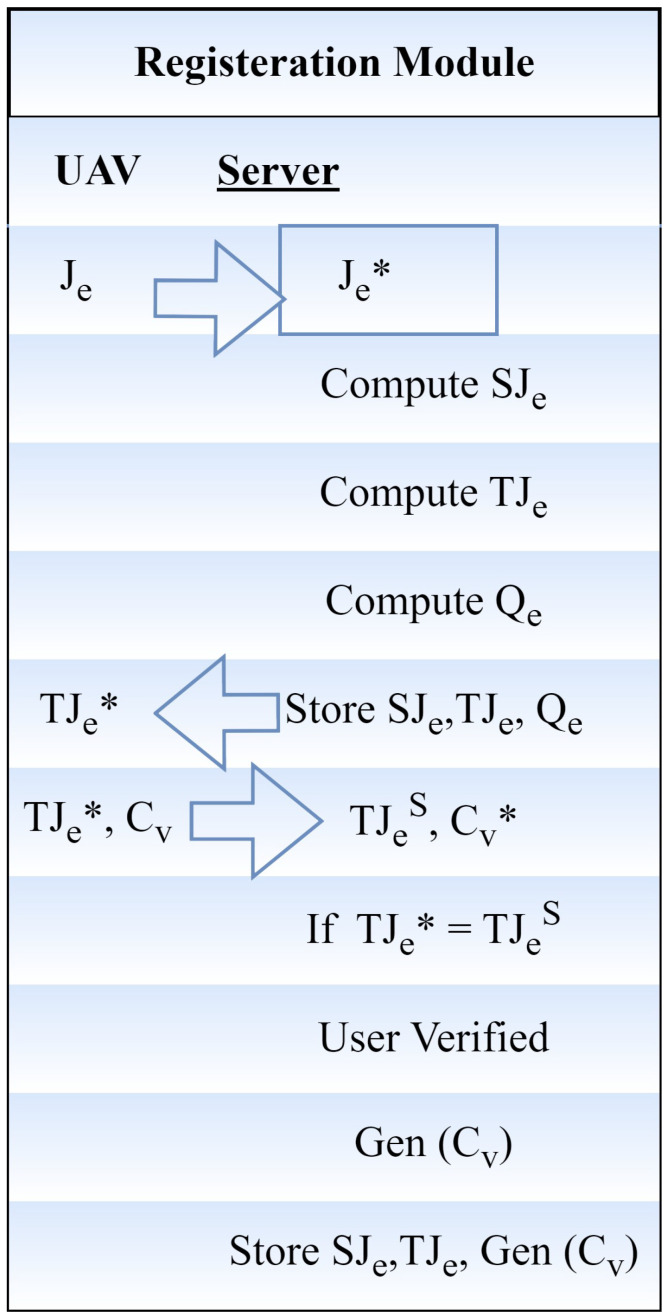
Pre-deployment module.

Following the completion of the user’s information submission to the server, the registration procedure is started. The first stage of the registration process is to register the user under the servers. Authentication is set up by registering the users and servers. The user registration step is shown as mathematical pseudo-code in Figure 3. This means that v, the user, has to perform these steps to safely register with server A:

1. The user selects an identity (J_v_) and securely sends a message to the server (A) to request registration. When the registration request is received, the pseudo-identity of the A computer user is described as
  (5) $$SJ_{v}=I\left(J_{v}*\right)mod\left(n\right)$$
  (6) $$TJ_{v}=F\left(J_{v}*\right)I\left(q<>S_{v}\phi L\right)$$
  J_v_ represents the received identity at the server side, S_v_ is the registration timestamp created by the user v, q is a master key, and n is the secret message.
  (7) $$L=n\phi I(J_{v}<>q_{v})$$
  when the user’s private key is represented by q_v_.
2. After securely sending the registration reply message to the user, server A saves the SJ_v_, TJ_v_, and Q_v_ in the UAV database. After the server generates the registration reply message, the user selects password B and enters biometric C_v_ at the sensor. The fuzzy extractor approach is used to complete biometric verification. The fuzzy extractor method makes use of a probabilistic generation function called Gen. This function accepts the user’s biometrics C_v_ as input and outputs the biometric secret key of length v bits as αj → 0, 1. The public parameter, H(C_v_), is defined as Gen(α). The information is then saved in the server’s memory. The user can still not use the server’s services, even if they have registered with the server unless the server authorizes them. The authentication procedure informs the server’s choice of the user’s permission to log.

#### 3.1.4. Login Module

The user must adhere to the precisely detailed procedures to complete the login phase. To begin, the user scans her fingerprint (C_v_) and enters her identification (J_v_) into the mobile device’s interface. Then, she identifies herself (C_v_) using the device’s sensor. If the mobile device has recently input biometrics C and the initial biometric from registration is known as the biometric key, it may calculate the key. To complete the assessment, the user v also determines the following parameters.

(8) $$z^{S}=I\left(J_{v}^{S}<>Z<>\beta^{S}\right)\phi \alpha^{S}$$

(9) $$a^{S}=I\left(T_{v}^{S}<>SJ_{v}<>\beta^{S}\right)\phi w$$

where the variable a^S^ stands for the received login request message and w is the master key.

(10) $$Z=F(SJ_{v}^{S}<>Q_{v}<>n)$$

(11) $$w=I\left(q<>n<>q_{v}\right)\phi S_{v}$$

The login request is calculated as

(12) $$z=I(J_{v}<>Z<>\beta )\phi \alpha$$

(13) $$a=I(TJ_{v}<>SJ_{v}<>\beta )\phi w$$

such that

(14) $$w=TJ_{v}*\phi X*$$

(15) $$w*=TJ_{v}^{S}\phi X$$

(16) s=w∗ϕX

The user sends a login request message to server A over a public channel, where Msg_1_ = (z, a, w, s, X).

#### 3.1.5. Authentication Module

The proposed model incorporates a two-level authentication scheme. Moreover, more than two levels of multifactor authentication results in a powerful security measure, but it does have some limitations when it comes to UAV-based secure data communication. Some of these limitations include the following:

1. *Connectivity*: UAVs may operate in areas with limited or no network connectivity, which can make it challenging to implement traditional MFA methods that rely on real-time communication with authentication servers.
2. *Payload limitations*: UAVs have payload limitations, which may restrict the types of hardware or software that can be carried onboard for MFA purposes.
3. *Environmental factors*: Environmental conditions such as extreme temperatures, high altitudes, and electromagnetic interference can impact the reliability and functionality of MFA components onboard UAVs.
4. *Power constraints*: UAVs have limited power sources, and running MFA processes may consume additional power, affecting the overall flight time and mission duration.

Upon receiving the login request from user v, the different processes are carried out by the user, server, and accessed UAV. In level 1, a session key is established by both the user and the UAV to ensure secure communication between them. Secure communication can only be achieved if the three parties involved, the user, the server, and the UAV, have been authorized. Once the login process for both the user and the server is complete, the authentication is carried out to initiate the connection. Various stages of verification are used to verify the users and the server to execute the authentication. The UAV starts by creating a random message N and sending it together with its associated parameters to the server for analysis. Once the server receives the message, it is assigned the value N. The messages N that were received are reviewed for verification. In level 2, the user receives the result after computing O_2_ and O_3_. On the user side, we obtain the request as S_r_. On the UAV side, we compute message O_4_, compare it to message O_1_, and finally provide the outcome. In addition to checking, the UAV makes sure that messages O_1_ and O_4_ are equivalent. If both are equivalent after verification, then communication may commence. Here, many layers of verification are carried out reciprocally to authenticate the users, and four distinct messages are used for the authentication process. The server verifies the user’s authorization before approving any communications. A variety of attacks may be countered by adjusting the hashing algorithm, which shows that the security protocol is resilient. Here are the steps for authentication, calculated and expressed as the following messages: N, O_1_, O_2_, O_3_, and O_4_.

(17) $$N=I(J_{e}<>TJ_{e}*)$$

(18) $$O_{1}=Nmod\left(h\right)$$

where h represents a random number.

(19) $$N^{\prime }=I\left(J_{e}*<>TJ_{e}\right)$$

(20) $$O_{2}=\left(\beta ,\alpha \right)mod\left(h\right)$$

(21) $$O_{3}=O_{1}\phi O_{2}$$

(22) $$O_{4}=O_{3}\phi O_{2}$$

The user’s authorization to access UAV data is granted upon completion of the fourth step of authentication. The UAV can obtain real-time information via its sensors and actuators after authentication and access control. In addition, as IoUAV devices do not need any kind of information exchange between themselves, the suggested layered authentication mechanism remains unaffected even if an attacker gains access to the UAV.

## 4. Experimental Validation

By contrasting the suggested approach with the current authentication and agreement technique, various metrics such as computation time, energy usage, packet loss, throughput, and latency are computed. Table 3 provides the simulated settings that were used to build the multilayer authentication technique. The PC used has a Windows 11 operating system, 16 GB of RAM, and an Intel i5 CPU running. For computational analysis, python SDK was used over the PC.

### 4.1. Simulation Attributes

The suggested multilayer authentication technique using the current technology is evaluated using the following metrics, namely *computation delay, energy consumption, packet loss, throughput, and latency.*

1. *Latency*: “Latency” refers to the overall amount of time it takes to broadcast the information. The delay is estimated in terms of computing time, energy usage, and packet loss.
  (23) $$Latency=\frac{Bit-count}{Transmission-rate}$$
2. *Throughput*: It describes the amount of data sent across a network at any given moment. Several variables compromise the communication system’s throughput. These include the limitations of the underlying analog physical media, the available computational capacity of the system’s components, and the end-to-end users. When various protocol overheads are taken into account, the data transfer rate falls short of the maximum throughput that may be achieved. Mathematically, consider the following:
  (24) $$Throughput=\frac{Packet-recieved-count}{Time}$$
3. *Loss of Packet*: *Packet loss* happens when data packets transmitted via a network do not arrive at their intended recipient. This might be because of transmission faults. Mathematically, consider the following:
  (25) $$Loss=\frac{Total-Packets-lossed}{Total-Packets}*100$$
4. *Energy Consumption*: It is the quantity of power that is used up to complete a specific job. To achieve energy efficiency, the energy consumption should be kept to a minimum.
  (26) Energy−Consumption=Power∗Delay
5. *Computation Delay*: The amount of time it takes to finish a whole operation is directly proportional to the number of rules that need to be applied.

### 4.2. Comparative  Analysis

With 150 nodes, 300 nodes, and 600 nodes, respectively, the current authentication and agreement approach is compared to the state-of-the-art technique presented by Perumalla et al. [33] in terms of computation time, energy consumption, packet loss, throughput, and latency as it is the most related work in the literature.

#### 4.2.1. One-Hundred-Fifty-Node Comparative Analysis

**Figure 4:**
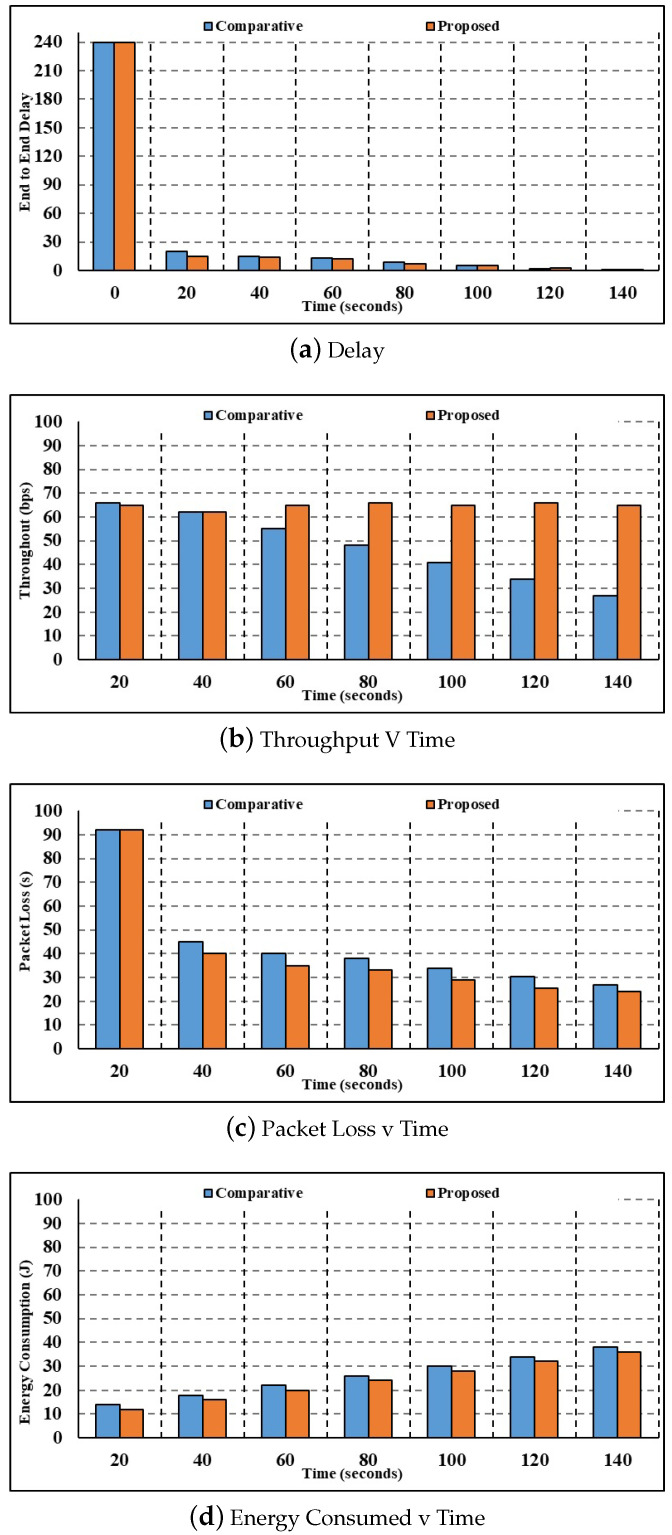
One-hundred-fifty-node comparative analysis.

Using 150 nodes, Figure 4 compares the current and suggested techniques in terms of latency, throughput, energy usage, packet loss, and calculation time.

1. Figure 4a depicts the results of the 150-node delay parameter investigation. When the time is 20 s, the current authentication method calculates a corresponding delay of 8.78 s, whereas the suggested multilayer authentication method computes a delay of 5.20 s. Compared to the state-of-the-art authentication and key agreement process, which takes 0.34 s, the proposed multilayer authentication process takes 0.08 s. Current authentication and key agreement have longer delays than the proposed multilayer authentication. Henceforth, the suggested multilayer authentication system has a very fast response time.
2. Figure 4b shows the results of the throughput parameter study with 150 nodes. While the throughput estimated using the current authentication and key agreement method is 61.60 bps for 40 s, the throughput estimated using the proposed multilevel authentication method is 64.20 bps. In comparison to the current authentication and key agreement throughput of 52.41 bps, the suggested multilayer authentication has a throughput of 62.74 bps for 100 s. The reduced throughput calculated by the suggested approach proves that the technique is very efficient at handling the data packets.
3. Figure 4c shows that the results of the investigation are expressed as a packet loss parameter with 150 nodes. At 60 s, the current authentication and key agreement method calculates a packet loss value of 3.72, whereas the proposed multilevel authentication method calculates a packet loss value of 2.66. In comparison to the current authentication and key agreement, the packet loss values calculated by the proposed multilayer authentication are 2.41. Minimal packet loss is an indication of how efficiently the suggested method processes packets and reduces packet loss.
4. Figure 4d shows the study in terms of the energy consumption parameter. Compared to the proposed multilayer authentication, which uses 16.79 J of energy for 100 s, the present authentication and key agreement uses 17.09 J. After 108 s, the suggested multilayer authentication uses 17.70 J of energy, compared to 19.63 J for the current authentication and key agreement. As a result, the suggested approach uses far less energy than the current one.

#### 4.2.2. Three-Hundred-Node Comparative Analysis

**Figure 5:**
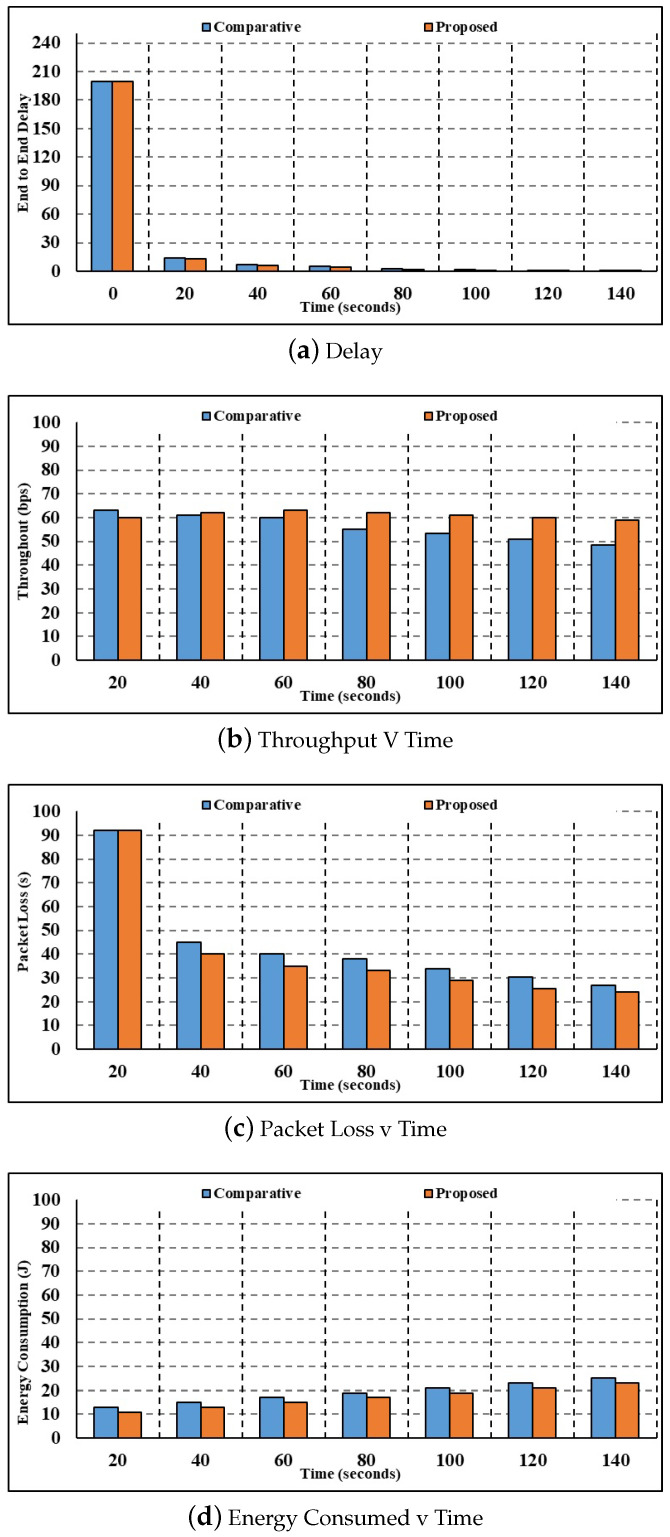
Three-hundred-node comparative analysis.

Figure 5 shows the results of an examination of current and suggested methodologies for latency, throughput, energy consumption, packet loss, and computation time measures with 100 nodes.

1. Figure 5a shows the results of the investigation for the delay parameter with 300 nodes. The current authentication and key agreement method calculate a corresponding delay value of 9.59 s for 20 s, whereas the proposed multilayer authentication method only takes 7.32 s. In comparison to the current authentication and key agreement delay of 1.46 s, the delay values calculated by the proposed multilayer authentication for 100 s are 1.38s.
2. Using 300 nodes, Figure 5b shows the throughput parameter analysis. The current authentication and key agreement method calculate a throughput value of 60.06 bps for 20 s, whereas the suggested multilayer authentication method achieves a throughput of 63.50 bps. In comparison to the current authentication and key agreement throughput of 48.21 bps, the throughput figures estimated by the proposed multilayer authentication for 100 s are 61.44 bps.
3. Figure 5c shows the results of the investigation with 300 nodes, broken down per packet loss parameter. At a time of 20 s, the packet loss calculated using the current authentication and key agreement method is 6.15 s, whereas the packet loss calculated using the suggested multilayer authentication method is 5.82 s. In comparison to the current authentication and key agreement, the packet loss values calculated by the proposed multilayer authentication for 100 s are 3.71 s.
4. Figure 5d shows the study in terms of the energy consumption parameter. Current authentication and key agreement use 16.03 J of energy for 100 s, while the suggested multilayer authentication consumes 17.11 J. Comparing the energy consumption of current authentication and key agreement at 17.55 J for 110 s to that of suggested multilayer authentication, the latter results in 15.45 J. As a result, the suggested approach uses far less energy.

#### 4.2.3. Six-Hundred-Node Comparative Analysis

**Figure 6:**
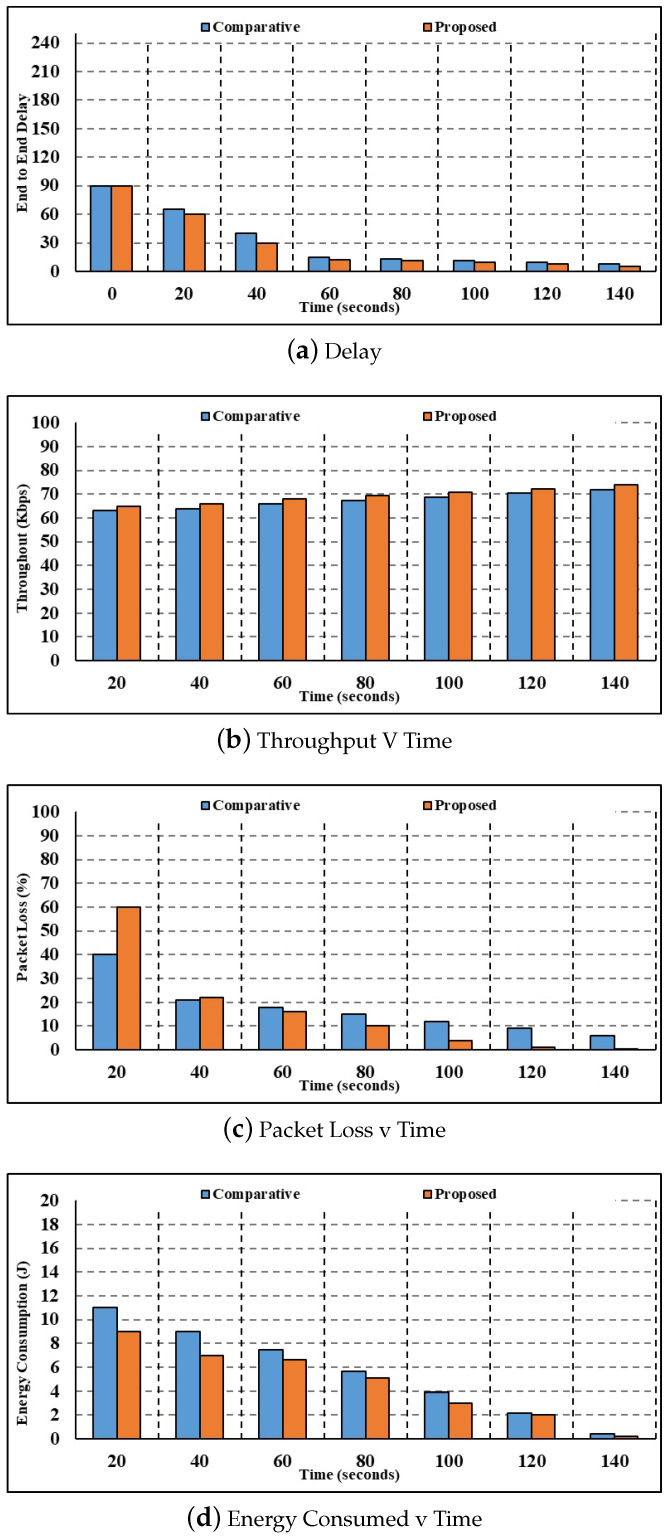
Six-hundred-node comparative analysis.

Figure 6 shows the results of a comparison of current and suggested approaches with 600 nodes, depending on factors such as computation time, energy consumption, packet loss, throughput, and latency.

1. Figure 6a illustrates the study on the delay parameter. The suggested multilayer authentication approach calculates a delay of 12.04 s when the time is 90 s, whereas the present method’s comparable delay is 16.46 s. The suggested multilevel authentication method calculates delay values of 8.45 s for 100 s, whereas the present authentication and key agreement method computes a delay of 14.73 s.
2. Figure 6b shows the results of the throughput parameter study with 600 nodes. There is a significant difference between the throughput values obtained using the current authentication and key agreement method (61.53 bps) and the suggested multilevel authentication method (63.73 bps) at a time of 22 s. Compared to the current authentication and key agreement throughput of 66.15 bps, the throughput values estimated by the proposed multilayer authentication are 68.34 bps for 75 s.
3. Figure 6c shows the results of the investigation with 600 nodes, broken down per packet loss parameter. There is a discrepancy between the packet loss values calculated using the current authentication and key agreement method (16.57 s) and the proposed multilevel authentication method (14.32 s) at a time of 55 s. Compared to the current authentication and key agreement, the packet loss values calculated by the proposed multilayer authentication are 10.01 s for 105 s. As a result of the suggested strategy’s fast processing of packets and mitigation of packet loss, the suggested technique has very little packet loss.
4. Figure 6d displays the results of the investigation concerning the energy consumption parameter. The current authentication and key agreement method uses 12.88 J of energy for 30 s, whereas the suggested multilayer authentication method uses 9.97 J. While current authentication uses 18.51 J of energy and key agreement uses 17.51 J for 80 s, the suggested multilayer authentication uses 17.51 J. Therefore, in comparison to the current way, the suggested strategy is superior and has less energy use.

### 4.3. Discussion

Table 4 presents a comparison of the proposed and current techniques, showcasing the essential agreement structure. The comparison is based on various metrics such as computation time, energy usage, packet loss, throughput, and latency. The objective is to minimize processing time, energy consumption, packet loss, and delay to achieve better results. The results show that the suggested multilayer authentication method outperforms the current authentication and key agreement approach in terms of maximum throughput with the least energy consumption, loss, and delay. The values attained for each measure for nodes 150, 300, and 600 are provided ahead. For 150 nodes, the suggested multilayer authentication method achieved a latency of 0.0958 s, while, for 300 nodes, it reached a value of 1.458 s. In contrast, the current authentication and key agreement approach revealed a larger delay of 1.365 s and 1.986 s while using 150 nodes and 300 nodes, respectively. The current technique has a throughput of 62.621 bps, whereas the proposed multilayer authentication scheme utilizing 150 nodes achieves 69.26 bps. Similarly, the current approach has a throughput of 50.262 bps, whereas the suggested multilayer authentication methodology utilizing 300 nodes achieves 56.654 bps. Using 150 nodes, the current technique calculates a packet loss rate of 4.586 s, whereas the proposed multilayer authentication scheme achieves a rate of 3.215 s. The current technique calculates a packet loss rate of 6.658 s with 300 nodes, while the suggested multilayer authentication scheme achieves a rate of 3.654 s. The suggested multilayer authentication strategy uses 20.265 J of energy compared to the current method’s energy consumption of 17.659 J when using 150 nodes. Similarly, the suggested multilayer authentication mechanism uses 15.265 J of energy compared to 18.255 J for the current method when using 300 nodes. The proposed multilevel authentication strategy performs better than the existing method, with an average minimum computational time of 3.45 s, a maximum throughput of 90.15 bps, a minimum energy consumption of 18.658 J, and a minimal packet loss of 8.854. Henceforth, this paper develops a multifactor authentication method to access UAV data to ensure secure communication before deployment, during login and authentication, and after deployment. Every UAV must be registered during the pre-deployment phase before it may be utilized, and, during registration, unique identifiers are exchanged between the server and the user. After the login phase, authentication is performed with minimal time, maximum throughput, and packet loss. However, in an attack scenario, the proposed model for managing and coordinating UAVs within an IoT framework may exhibit certain vulnerabilities and behaviors. Several possible solutions can be incorporated into the proposed approach:

1. *Denial of Service (DoS) Attacks*: Attackers may attempt to overwhelm the system with a high volume of illegitimate requests, disrupting communication and coordination. This could potentially lead to a loss of control over UAVs and operational disruptions. Implementing rate limiting, traffic filtering, and employing redundant communication channels can help to mitigate the impact of DoS attacks and ensure continuity of operations.
2. *Man-in-the-Middle (MitM) Attacks*: Unauthorized entities could intercept and manipulate communication between UAVs and the IoT system, compromising data integrity and security. Implementing end-to-end encryption, digital signatures, and mutual authentication can help to prevent and detect MitM attacks, ensuring the integrity and confidentiality of data transmission.
3. *Unauthorized Access*: Robust authentication mechanisms should be in place to prevent unauthorized access attempts, such as strong user authentication and access control policies. Implementing multifactor authentication, role-based access control, and regular security audits can help to prevent unauthorized access and protect sensitive information.
4. *Data Tampering*: To prevent data tampering, data integrity checks, cryptographic hashing, and secure transmission protocols should be employed to detect and prevent unauthorized modifications to transmitted data. Implementing data validation mechanisms, digital signatures, and secure communication protocols can help to ensure the integrity of data transmitted between UAVs and the IoT system.
5. *Identity Spoofing*: Employing strong identity verification methods and secure communication channels can help to prevent identity spoofing attacks. Implementing certificate-based authentication, biometric authentication, and secure token exchange can help to mitigate the risk of identity spoofing attacks.
6. *Registry Manipulation*: Implementing secure registry management practices, such as access controls, audit trails, and tamper-evident logging, can help to prevent unauthorized manipulation of the IoUAV registry. Employing secure registry management practices, such as access controls, audit trails, and regular integrity checks, can help to prevent unauthorized manipulation of the IoUAV registry.

### 4.4. Security Validation

This section discusses the security validation of the proposed model concerning the state-of-the-art works of [13,33,34]. The security analysis is performed for the various types of attacks performed using an online dataset Source: https://www.stratosphereips.org/datasets-iot23 (accessed on 16 January 2024). The results are computed and compared for statistical measures of specificity, sensitivity, accuracy, and F-measure. Moreover, numerous error rates have been computed, including root mean square error, root absolute error, and mean absolute error. Table 5 depicts the computed results. The proposed model acquires a precision of 94.46% across different attack datasets, outperforming [13] (88.44%), ref. [34] (89.23%), and [33] (91.27%). In terms of specificity, the provided model achieves a value of 96.97%, surpassing [13] (87.02%), ref. [34] (89.22%), and [33] (92.33%). Sensitivity analysis shows that the suggested model achieves a high value of 96.54%, outperforming [13] (86.49%), ref. [34] (89.64%), and [33] (91.95%). Further details and computational analysis can be found in Table 5, indicating the superiority of the proposed technique, making it suitable for the current scenario.

### 4.5. Limiting Aspects

The proposed model for managing and coordinating UAVs through an IoT system seems to address the critical issue of security in IoUAV communication. However, there are several potential limitations and drawbacks to consider:

1. Scalability: The scalability of the proposed model may not have been thoroughly addressed. As the number of UAVs and users increases, the system’s ability to handle a larger load of registrations, logins, and authentications may become a challenge.
2. Connectivity and Latency: The model’s effectiveness may be limited in scenarios with intermittent connectivity or high latency as real-time communication and authentication could be compromised.
3. User Experience: The user registration and login process may introduce complexities for UAV operators, potentially affecting the overall user experience and operational efficiency.
4. Maintenance and Updates: The long-term maintenance and update process for the IoUAV registry and authentication modules should be considered to ensure ongoing security and reliability.

#### Uav-Related Limiting Aspects

While the proposed model addresses security concerns in UAV communication through a layered authentication approach, there are various other factors to consider for a comprehensive UAV model, such as:

1. *Flight Performance*: The model should account for the specific flight performance requirements of UAVs, including stability, maneuverability, and payload capacity.
2. *Regulatory Compliance*: Compliance with aviation regulations, airspace restrictions, and licensing requirements is crucial for UAV operations.
3. *Environmental Adaptability*: The model should consider the adaptability of UAVs to diverse environmental conditions, including weather, temperature, and terrain.
4. *Mission-specific Considerations*: Addressing the unique requirements of different UAV missions, such as surveillance, mapping, delivery, or search and rescue.

### 4.6. Application Deployment Scenarios

The proposed model for managing and coordinating UAVs through an IoT system would be ideal for deployment in various applications and environments. Some of the ideal applications and environments for the deployment of this model include the following:

1. *Industrial Sector*: The model can be deployed in industrial environments where UAVs are used for tasks such as infrastructure inspection, inventory management, and surveillance. The security and coordination provided by the proposed model can enhance operational efficiency and safety in industrial settings.
2. *Emergency Response*: Deploying the model in emergency response scenarios, such as search and rescue operations or disaster management, can enable secure and coordinated communication among UAVs and ground personnel, facilitating effective response efforts.
3. *Agriculture*: In agricultural applications, the model can be utilized for coordinating UAVs involved in crop monitoring, pesticide spraying, and irrigation management. The secure communication facilitated by the model can help to optimize agricultural operations.
4. *Urban Planning and Infrastructure Development*: The proposed model can be deployed in urban planning and infrastructure development projects where UAVs are used for surveying, mapping, and construction monitoring. It can ensure secure data transmission and coordination among multiple UAVs involved in such projects.
5. *Remote Sensing and Environmental Monitoring*: Environments such as remote sensing missions, environmental monitoring, and wildlife conservation can benefit from the secure communication and coordination provided by the proposed model, enabling effective data collection and analysis by UAVs.

In these application areas, the proposed model’s layered authentication approach can contribute to enhanced security, efficient coordination, and real-time data transmission, thereby improving the overall effectiveness of UAV operations within an IoT framework.

## 5. Conclusions

The novel contribution of this work is to propose a two-factor authentication method that can be implemented by both users and UAVs through a server connection. Before obtaining the session key, both the user and the UAV must authenticate each other to protect against various forms of attacks on secure communication. The authentication process utilizes the produced key during the authentication step to carry out the security analysis. To prevent unauthorized access to data and produce real-time data, the user must log in to the UAV. The suggested approach provides advanced features with enhanced security and is successful in computing and communication. The proposed technique surpasses other current solutions with its maximum throughput of 90.15%, minimum latency of 3.255 s, and packet loss of 8.854%. It outperforms the current authentication and key agreement system by 72.23%, 1.23%, 15.45%, 5.86%, and 14.46% for 600 nodes when measuring computing time, energy consumption, packet loss, throughput, and latency, respectively. Secure communication is a crucial component of the suggested approach, which is why it provides cutting-edge functionalities for this purpose. Future research will focus on assessing cyber vulnerabilities in IoUAV by exploring potential scenarios. Additionally, we will create a system that uses blockchain technology to control access to IoUAV, enabling secure communication between them. 

## Figures and Tables

**Table 1:** State-of-the-art comparison (1 available, 0 not available).

References	[29]	[30]	[31]	[32]	[23]	[22]	[33]	Proposed
UAV	1	1	1	1	1	1	1	1
Data Analysis	0	0	0	0	0	0	1	1
Data Repository	1	1	1	1	0	0	1	1
Quantification	1	1	1	1	1	0	0	1
Security	1	1	1	1	1	1	1	1
Authentication	0	0	0	0	0	1	0	1
IoT	1	1	1	1	1	1	1	1
Numerical	0	1	1	0	1	0	0	1
Latency	0	0	0	0	0	1	1	1
Energy Efficacy	0	0	0	0	0	0	0	1

**Table 2:** Parameters and description.

Symbolic Representation	Description
e	UAV node
A	Server Module
n	Secret Message
J	Identity Value
SJ	Relative Identity Value
T	Server Module
Q	Polynomial Identity Value
S	Registration Timestamp
v	User
q	Private Key
H	Public attribute
C	Fingerprint
h	Random Number
O	Output measures

**Table 3:** Simulation attributes.

S. No.	Attribute	Measure
1	Energy	25 J
2	Power (Transmission)	0.833 W
3	Power (Receiving)	0.0637 W
4	Power (Idle)	0.064 W
5	Power (Sense)	0.0164 W

**Table 4:** Comparative analysis.

Metric	Node Count	State-of-the-Art Technique	Proposed Technique
Latency	150	1.365	0.958
Latency	300	1.986	1.458
Latency	600	3.995	3.255
Throughput	150	62.621	69.26
Throughput	300	50.262	56.654
Throughput	600	79.265	90.15
Loss of Packet	150	4.586	3.215
Loss of Packet	300	6.658	3.654
Loss of Packet	600	10.245	8.854
Consumed Energy	150	20.265	17.659
Consumed Energy	300	18.255	15.265
Consumed Energy	600	20.999	18.658
Computation Delay	150	2.958	2.82
Computation Delay	300	2.145	1.92
Computation Delay	600	3.958	3.45

**Table 5:** Statistical efficiency.

Parameter	[13]	[34]	[33]	Proposed
**Precision**	88.44%	89.23%	91.27%	94.46%
**Specificity**	87.02%	89.22%	92.33%	96.97%
**Precision**	87.44%	89.46%	91.07%	94.56%
**Sensitivity**	86.49%	89.64%	91.95%	96.54%
**F-Measure**	86.96%	89.26%	90.45%	95.69%
**Root Relative Squared Error (RRSE)**	6.67 ± 0.38%	6.64 ± 0.68%	6.64 ± 0.33%	2.23 ± 0.66%
**Root Mean Square Error (RMSE)**	6.42 ± 0.03%	5.03 ± 0.37%	4.89 ± 0.23%	2.35 ± 0.37%
**Relative Absolute Error (RAE)**	5.66 ± 0.43%	6.63 ± 0.66%	7.21 ± 0.19%	4.11 ± 0.22%
**Mean Absolute Error (MAE)**	6.09 ± 0.03%	7.22 ± 0.72%	7.78 ± 0.27%	4.43 ± 0.42%

## Data Availability

The data used to support the findings of this study are available from the corresponding author upon request.
